# Mechanisms of *mepA* Overexpression and Membrane Potential Reduction Leading to Ciprofloxacin Heteroresistance in a *Staphylococcus aureus* Isolate

**DOI:** 10.3390/ijms26052372

**Published:** 2025-03-06

**Authors:** Mengyuan Li, Qianting Jian, Xinyi Ye, Mou Jing, Jia’en Wu, Zhihong Wu, Yali Ruan, Xiaoling Long, Rongmin Zhang, Hao Ren, Jian Sun, Yahong Liu, Xiaoping Liao, Xinlei Lian

**Affiliations:** 1 State Key Laboratory for Animal Disease Control and Prevention, South China Agricultural University, Guangzhou 510642, China; mengyuan_0217@163.com (M.L.); jqianting@163.com (Q.J.);; 2 National Risk Assessment Laboratory for Antimicrobial Resistance of Animal Original Bacteria, South China Agricultural University, Guangzhou 510642, China; 3 Guangdong Provincial Key Laboratory of Veterinary Pharmaceutics Development and Safety Evaluation, South China Agricultural University, Guangzhou 510642, China

**Keywords:** *Staphylococcus aureus*, heteroresistance, ciprofloxacin, MRSA, limonene, membrane potential

## Abstract

Heteroresistance has seriously affected the evaluation of antibiotic efficacy against pathogenic bacteria, causing misjudgment of antibiotics’ sensitivity in clinical therapy, leading to treatment failure, and posing a serious threat to current medical health. However, the mechanism of *Staphylococcus aureus* heteroresistance to ciprofloxacin remains unclear. In this study, heteroresistance to ciprofloxacin in *S. aureus* strain 529 was confirmed by antimicrobial susceptibility testing and population analysis profiling (PAP), with the resistance of subclonal 529_HR based on MIC being 8-fold that of the original bacteria. A 7-day serial MIC evaluation and growth curves demonstrate that their phenotype was stable, with 529_HR growing more slowly than 529, but reaching a plateau in a similar proportion. WGS analysis showed that there were 11 nonsynonymous mutations and one deletion gene between the two bacteria, but none of these SNPs were directly associated with ciprofloxacin resistance. Transcriptome data analysis showed that the expression of membrane potential related genes (*qoxA*, *qoxB*, *qoxC*, *qoxD*, *mprF*) was downregulated, and the expression of multidrug resistance efflux pump gene *mepA* was upregulated. The combination of ciprofloxacin and limonene restored the 529_HR MIC from 1 mg/L to 0.125 mg/L. Measurement of the membrane potential found that 529_HR had a lower potential, which may enable it to withstand the ciprofloxacin-induced decrease in membrane potential. In summary, we demonstrated that upregulation of *mepA* gene expression and a reduction in membrane potential are the main heteroresistance mechanisms of *S. aureus* to ciprofloxacin. Additionally, limonene may be a potentially effective agent to inhibit ciprofloxacin heteroresistance phenotypes.

## 1. Introduction

*Staphylococcus aureus* is one of the most well-known opportunistic pathogens, causing infections in humans and animals ranging from relatively mild skin and soft tissue infections to serious life-threatening diseases such as bacteremia, infective endocarditis, osteomyelitis, and pneumonia [1,2]. Of the primary pathogens linked to medication resistance in 2019, *S. aureus*, a member of the ESKAPE pathogen group, came in second place. Methicillin-resistant *S. aureus* (MRSA) infections produce higher morbidity and mortality than methicillin-sensitive *S. aureus* (MSSA) infections. MRSA caused over 100,000 fatalities due to antimicrobial resistance in 2019 [3]. Currently, vancomycin, daptomycin, ceflorin, and linezolid are the most commonly used antibiotics for treating MRSA infections [4]. However, the abuse of antibiotics and the evolution of bacteria have led to a progressive rise in *S. aureus* resistance in recent decades. With the increasing resistance of these bacteria to the antibiotics above, the investigation of alternative antimicrobial treatments is necessary. Fluoroquinolones have been mentioned as potentially effective medications for the treatment of MRSA [5], among which ciprofloxacin is a substitute for vancomycin for MRSA [6].

Ciprofloxacin exhibits broad antibacterial activity against both Gram-positive and Gram-negative bacteria [7]. However, the continual use of fluoroquinolone can result in therapeutic failure against *S. aureus*. The primary mechanism of fluoroquinolone resistance in *S. aureus* involves mutations in target genes for DNA helicase and topoisomerase IV, as well as overexpression of efflux pumps [8]. Mutations in genes such as *gyrA* and *parC* can lead to ciprofloxacin resistance in *S. aureus* [9]. Additionally, increased expression of multidrug-resistant efflux pump genes including *norA* and *sdrM* in the MFS family and *mepA* in the MATE family reduces the sensitivity of strains to ciprofloxacin [10,11]. It has been discovered that limonene functions as an efflux pump inhibitor to prevent the expression of the *mepA* efflux pump [12].

Heteroresistance refers to the phenomenon that there is a small part of a resistant subpopulation in a single isolated sensitive strain, with MIC >8-fold higher than the main population [13]. This phenomenon impedes antibiotic therapeutic evaluation of pathogenic bacteria, causing misjudgment of antibiotic sensitivity in clinical therapy and leading to treatment failure, posing a serious threat to current medical health. The prevalence of heteroresistant vancomycin-intermediate *S. aureus* increased 10-fold in 10 years, indicating serious and unavoidable clinical challenges for infection control and treatment [14,15]. Heteroresistance of *S. aureus* to antibiotics like daptomycin [16], omacycline [17], and illavacycline [18] has also been reported. However, heteroresistance of *S. aureus* to ciprofloxacin has not been reported. Heteroresistance poses challenges in detection and study due to phenotypic and genetic instability [13,19]. There are relatively few reports of heteroresistance, and the mechanism is not fully elucidated, requiring further exploration.

Gene expression heterogeneity is common in bacterial populations, with distinct bacteria exhibiting distinct transcriptional and metabolic patterns [20,21]. Membrane potential represents a free energy source regulating wide-ranging bacterial physiology and behavior, including transmembrane transport, flagellar movement, and antibiotic resistance [22,23]. Bacteria can respond to stress by regulating the flux of ions across the cell membrane, resulting in changes in the cell membrane potential. Additionally, measuring membrane potential can help determine whether a bacterial subpopulation is highly or poorly susceptible to antibiotics targeting ribosomes [24]. Some studies suggest that reduced metabolic activity stemming from decreased membrane potential may lead to persistence [25]. A decrease in membrane potential may reduce antibiotic sensitivity [26]; the electric potential (ΔΨ) component can be considered a potential antibacterial target.

In this study, we discovered that a clinical isolate of *S. aureus* GD18_SA_529 from the Guangdong Provincial Center for Disease Control and Prevention was heteroresistant to ciprofloxacin. Furthermore, we identified a subpopulation of the ciprofloxacin heteroresistant isolate and investigated the underlying mechanism of the heteroresistant phenotype.

## 2. Results

### 2.1. The Ciprofloxacin Heteroresistance of Staphylococcus aureus 529

**Figure 1:**
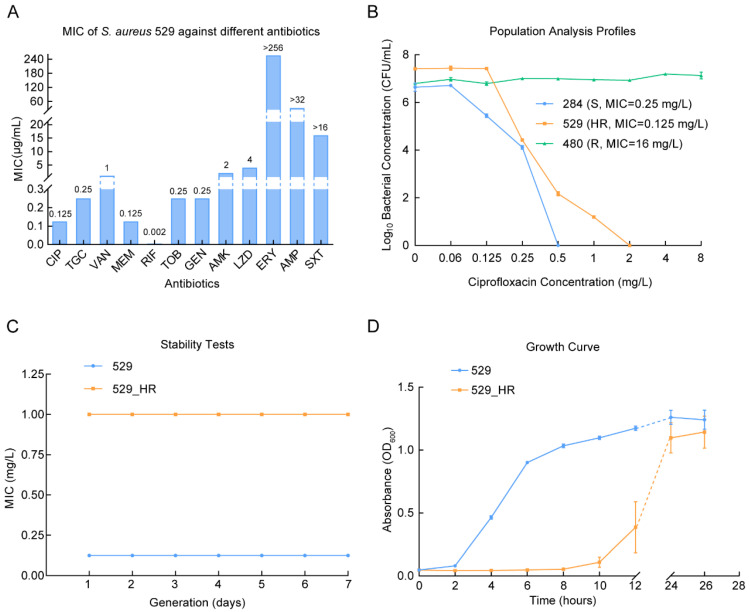
Verification of ciprofloxacin heteroresistance in *S. aureus* 529. (**A**) MIC of *S. aureus* 529 against different antibiotics. Abbreviations: CIP, ciprofloxacin; TGC, tigecycline; VAN, vancomycin; MEM, meropenem; RIF, rifampicin; TOB, tobramycin; GEN, gentamycin; AMK, amikacin; LZD, linezolid; ERY, erythromycin; AMP, ampicillin; SXT, trimethoprim/sulfamethoxazole. (**B**) Population analysis assays of the *S. aureus* 529, 284, 480 strains using ciprofloxacin. (**C**) Stability tests of the parental strain *S. aureus* 529 and heteroresistant subclone *S. aureus* 529_HR. (**D**) Growth curves of *S. aureus* 529 and 529_HR.

Antimicrobial susceptibility testing identified that *S. aureus* 529 was resistant to penicillin, macrolides, and sulfonamides. Conversely, it was not resistant to fluoroquinolones, aminoglycosides, beta-lactam, glycopeptides, glycylcyclines, oxazolidinones, and rifamycin antibiotics (Figure 1A). Interestingly, this strain was below the breakpoint of resistance to ciprofloxacin (MIC = 0.125 mg/L). However, PAP confirmed that this strain had a heteroresistant phenotype to ciprofloxacin, which could grow at 8-fold of the MIC ciprofloxacin concentration (1 mg/L) (Figure 1B). The 8-fold MIC ciprofloxacin heteroresistant subclone was isolated and named 529_HR. Furthermore, it was determined that the MBCs of 529 and 529_HR were 0.25 mg/L and 2 mg/L, respectively (Table S2). Both 529 and 529_HR established a stable MIC of ciprofloxacin after passaging on ciprofloxacin-free culture medium for 7 days (Figure 1C). By comparing the growth curves of 529 and 529_HR, it was discovered that the growth of 529_HR was slower than that of 529, but the proportion of bacteria that reached the plateau stage was comparable (Figure 1D). By calculating the specific growth rates of 529 and 529_HR during their respective logarithmic growth phases, it was determined that the specific growth rate of 529 was 0.205 and that of 529_HR was 0.139. This suggests the metabolic activities of these two strains derived from a single ancestor are different.

### 2.2. Genomic Characteristics Between 529 and 529_HR

To investigate the mechanism by which *S. aureus* strain 529 produces a heteroresistant phenotype, we conducted whole-genome sequencing of 529 and 529_HR. We obtained assembled genomes of 529 and 529_HR (N50_529_ = 174,991 and N50_529_HR_ = 174,974), which annotated 2648 and 2647 genes, respectively. The total obtained number of annotated genes was comparable to the normal strain of *S. aureus* ATCC29213, which has 2586 genes. Each has a ~2.8 Mb genome containing two plasmids, pSJH101 and pMW2. The β-lactam resistance gene *blaZ*, macrolide resistance gene *erm(A)*, aminoglycoside resistance gene *spc,* and rifamycin resistance gene *rpoB* exist in the genome. In addition, the genome also contains *sdrM* of the multi-resistance efflux pump MFS family and *mepA* of the MATE family (Table 1). Only a few variations were observed across the strains, including 11 nonsynonymous SNPs and a deletion (Table 2). Surprisingly, neither the SNPs nor the deletion genes were directly associated with resistance to ciprofloxacin. Of these nonsynonymous mutation sites, *clfB* is a member of the MSCRAMM (Microbial Surface Component Recognition Adhesive Matrix molecules) family of surface proteins, described as fibrinogen binding agglutination factors, *asd* is aspartate-semialdehyde dehydrogenase, which participates in the synthesis of cell wall components such as diaminopimelate, *ebh* is a hyperosmolarity resistance protein, which is an adhesion protein associated with cell walls, *ssl3* is staphylococcal superantigen-like protein 3, which participates in immune regulation, and *farB* encodes the fatty acid resistant protein FarB. None of these mutations were associated with ciprofloxacin resistance, so genetic mutations do not contribute to understanding the mechanism of heteroresistance.

### 2.3. Transcriptional Regulation of the Heteroresistant Subclone 529_HR

**Figure 2:**
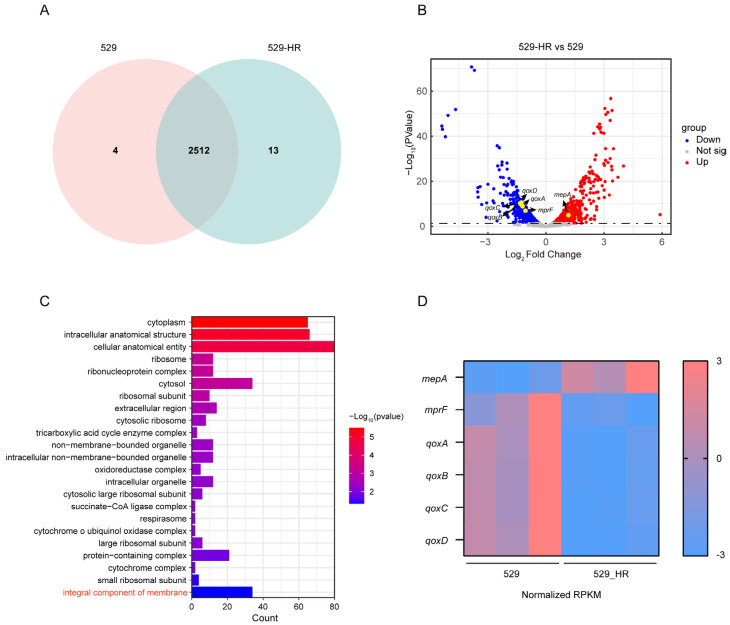
Transcriptional regulation of *S. aureus* 529 and 529_HR. (**A**) Venn diagram of expressed genes in 529 and 529_HR. (**B**) Volcano plot of 529_HR vs. 529. The blue dot on the volcano plot indicates down-regulated genes (Log_2_ Fold change ≤ 0 and FDR < 0.05). The red dot on the volcano plot indicates up-regulated genes (Log_2_ Fold change ≥ 0 and FDR < 0.05). The genes labeled by the yellow plot are genes linked to membrane potential. (**C**) The chromosomal DEGs GO enrichment analysis in cellular components of 529_HR vs. 529. (**D**) Regulation of genes linked to membrane potential (*mepA*, *mprF*, *qoxA*, *qoxB*, *qoxC*, *qoxD*).

The transcriptional profiles of 529 and 529_HR were obtained via transcriptome sequencing. In total, 2516 genes were expressed in strain 529, while 2525 genes were expressed in strain 529_HR (Figure 2A). A total of 497 significant DEGs (differentially expressed genes) were identified between 529_HR and 529, including 301 up-regulated genes and 196 down-regulated genes (Figure 2B). GO cellular component analysis revealed that integral components of the membrane, intracellular anatomical structure, and cytochrome o ubiquinol oxidase complex were altered significantly (Figure 2C). Enriched biological process terms included metabolic processes such as organic acid catabolic process and amino acid catabolic process. Additionally, DEGs were enriched in molecular function terms including oxidoreduction-driven active transmembrane transporter activity, oxidoreductase activity, structural constituents of ribosomes, and so on (Figure S1). Notably, we observed down-regulation of genes associated with membrane potential (*qoxA*, *qoxB*, *qoxC*, *qoxD*, *mprF*) and up-regulation of the multidrug resistance efflux pump *mepA* among genes implicated in the integral component of the membrane term (Figure 2D and Figure S2). This suggests that the ciprofloxacin resistance of 529_HR may be associated with the up-regulated expression of *mepA* and altered membrane potential. Furthermore, the expression of *mepA*, *qoxA*, *qoxB*, and *qoxC* were validated by RT-qPCR (Figure S3). Moreover, the transcriptional levels of *gyrA*, *gyrB*, *parC*, and *parE* in strain 529_HR vs. strain 529 showed no statistically significant changes (log_2_ fold-change values: −0.47, −0.35, −0.70, −0.77, respectively; all magnitudes < 1). These results suggest that the expression of topoisomerase/gyrase-related genes is not substantially altered in strain 529-HR.

### 2.4. Limonene Changes the Heteroresistance Phenotype of 529

**Figure 3:**
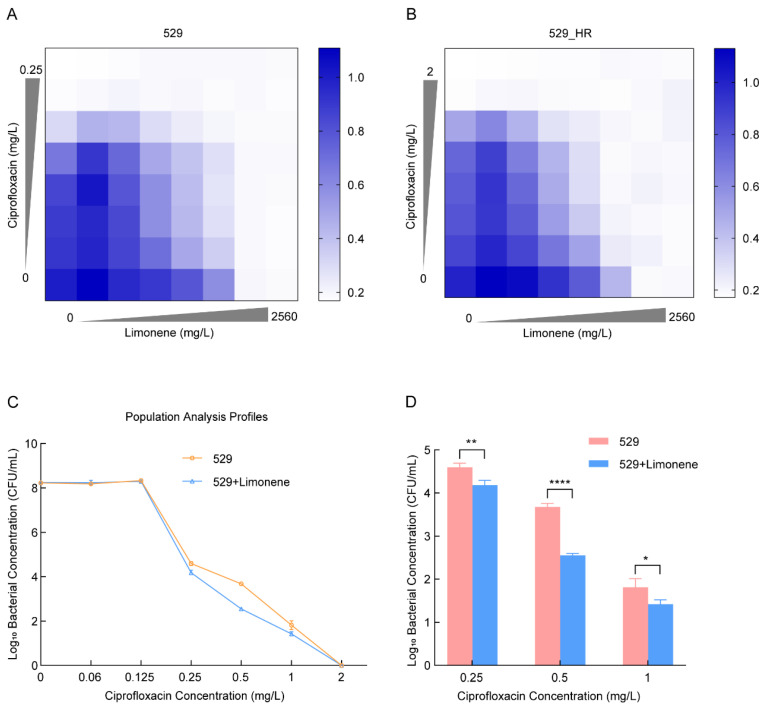
Limonene combined with ciprofloxacin inhibited the heteroresistance phenotype of 529_HR. (**A**,**B**) Limonene combined with ciprofloxacin inhibited 529 and 529_HR at different concentrations. (**C**) Population analysis assays of *S. aureus* 529 with and without limonene using ciprofloxacin. (**D**) The amount of surviving 529 bacteria with and without limonene at 0.25, 0.5, and 1 mg/L ciprofloxacin. Note: * *p* < 0.05, ** *p* < 0.01, **** *p* < 0.0001.

**Figure 4:**
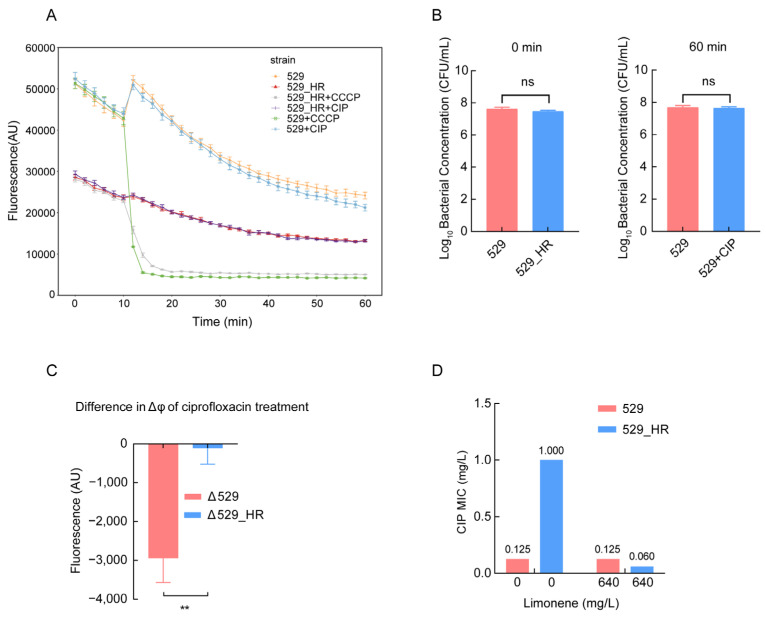
Changes in membrane potential lead to changes in the sensitivity of 529_HR to ciprofloxacin. (**A**) Strains 529 and 529_HR were continuously monitored for fluorescence for 1 h with and without ciprofloxacin and CCCP. Abbreviation: CCCP, carbonyl cyanide m-chlorophenyl hydrazine. (**B**) The amount of bacteria in 529 and 529_HR at 0 min and after 60 min of ciprofloxacin treatment. (**C**) ΔΨ values of 529 and 529_HR after ciprofloxacin treatment. (**D**) MIC values of 529 and 529_HR for ciprofloxacin at 0 mg/L and 640 mg/L limonene concentrations. Note: “ns” *p* ≥ 0.05, ** *p* < 0.01.

We utilized limonene to inhibit MepA efflux pump expression to confirm that *mepA* overexpression is a cause of *S. aureus*’s heteroresistance to ciprofloxacin. Combining ciprofloxacin and the efflux pump inhibitor limonene revealed 640 mg/L as the optimal concentration for inhibiting 529_HR (Figure 3A,B). At this concentration, 529_HR’s MIC to ciprofloxacin decreased from the original 1 mg/L to 0.06 mg/L (Figure 4D), restoring sensitivity to ciprofloxacin without affecting 529’s MIC. It was established that the up-regulation of *mepA* expression was one of the reasons for the increase in MIC in 529_HR. Thus, limonene may be a potential inhibitor of 529’s heteroresistance phenotype. PAP experiments showed limonene could indeed inhibit 529_HR’s resistant phenotype to some extent (Figure 3C). Treated with limonene and ciprofloxacin, 529’s surviving subpopulations significantly decreased, dropping 2.6, 13.5, and 2.6-fold under 0.25, 0.5, and 1 mg/L ciprofloxacin, respectively (Figure 3D). However, heteroresistance could not be completely suppressed (Figure 4D). These results indicate that up-regulated *mepA* is not the sole mechanism of heteroresistance.

### 2.5. Reduced Membrane Potential Led to Heteroresistance

Analysis of the transcriptome results showed downregulation of membrane potential-related genes (*qoxA*, *qoxB*, *qoxC*, *qoxD*, *mprF*) in 529_HR (Figure 2D). These genes’ transcriptional regulation may reduce their membrane potential. Using a DiOC2(3) membrane potential fluorescence probe, the membrane potentials of 529 and 529_HR were measured. The latter was significantly lower, with a nine-fold reduction compared to the former (Figure 4C), consistent with transcriptome analysis findings. Moreover, 529’s membrane potential dropped dramatically with four-fold or higher MIC ciprofloxacin concentrations, but 529_HR’s remained relatively unchanged (Figure 4A and Figure S4). Plate counting showed no significant difference in viable bacteria between 529 and 529_HR at 0 min and no significant difference in 529 after 60 min ciprofloxacin treatment (Figure 4B). This suggests neither inconsistent bacterial quantity nor death caused the membrane potential change in 529 and 529_HR. The findings show 529_HR has a low potential, enabling it to withstand the ciprofloxacin-induced membrane potential decrease.

## 3. Discussion

Heteroresistance poses great challenges for evaluating antibiotic therapeutic effects on pathogenic bacteria, with clinically detected minimum inhibitory concentrations sometimes failing to inhibit pathogen growth, resulting in treatment failure and threatening modern medicine safety. Heteroresistance may also be an intermediate step towards developing resistance [27]. Fluoroquinolones like ciprofloxacin, an alternative to vancomycin, can treat MRSA infections. However, at present, there are few reports on the heteroresistance of *S. aureus* to fluoroquinolones. In this study, the mechanism of heteroresistance of *S. aureus*, belonging to the ESKAPE group, to ciprofloxacin was mainly explored.

Firstly, in this study, we identified a ciprofloxacin heteroresistant *S. aureus* strain 529, with both the parent strains and resistant subclone (529_HR) exhibiting stable inheritance, consistent with prior findings by Chuanzhen Zhang et al. [28]. Additionally, the growth rate of the resistant subclone was slower compared to the parent strain. Previous studies have linked low growth in *Acinetobacter baumannii* to colistin heteroresistance [29]. Similarly, vancomycin-heteroresistant *S. aureus* strains, including heteroresistant vancomycin-intermediate *S. aureus* (hVISA) and vancomycin-intermediate *S. aureus* (VISA) exhibited slower growth rates relative to fully sensitive strains [15]. Consequently, heteroresistance-related recurrent and persistent infections may stem from this phenomenon.

Many studies have shown that mutations, insertions, or deletions of genes associated with antibiotic resistance can lead to ciprofloxacin resistance, such as *gyrA*, *parC*, and *rsxC* mutations causing heteroresistance of *Salmonella* to ciprofloxacin [28]. Some reports have indicated that the high prevalence of heteroresistance in *S. aureus* is attributed to a large number of mutations occurring in core genes [30]. However, SNP analysis of the 529 and 529_HR genomes found none of their SNPs were associated with these genes. Our data exclude transcriptional dysregulation of DNA topoisomerases/gyrase as a determinant of ciprofloxacin heteroresistance in *S. aureus* 529_HR. Instead, mutations were found in genes such as fibrinogen binding agglutination factor, extracellular matrix binding protein, aspartate hemialdehyde dehydrogenase, and staphylococcal superantigen 3. These genes play different roles in different physiological activities of bacteria, such as encoding fatty acid resistance proteins and participating in immune regulation. It is worth noting that most of these mutated genes are related to bacterial cell wall formation, such as *asd* participating in the synthesis of cell wall components such as diaminopimelate, and *ebh* being an adhesion protein associated with cell walls. It has been reported that cell wall thickness in bacteria can lead to heteroresistance [31]. However, none of these genes were associated with cell wall thickening. Therefore, as none of these changes were linked to ciprofloxacin resistance nor cell wall thickening, DNA mutations could not explain the mechanism underlying *S. aureus*’s heteroresistance to ciprofloxacin.

Many studies have suggested that MATE family efflux pumps, including *mepA*, are associated with ciprofloxacin resistance [32,33]. We investigated the potential role of *mepA* in the mechanism of ciprofloxacin heteroresistance in *S. aureus* and found that *mepA* expression level in the ciprofloxacin heteroresistant subgroup was significantly higher than that in the parental strains. These results suggested that increased *mepA* expression might be a cause of heteroresistance.

Limonene is a secondary metabolite in phytochemistry, found in essential oil compositions of aromatic plants like Micrantha and tarragon [12]. It has been reported to inhibit the activity of *S. aureus* efflux pumps [34,35]. Freitas’ studies have shown that limonene can efficiently fit into the MepA structure and suppress MepA efflux systems, inhibiting bacterial resistance [12]. In this study, limonene decreased the MIC of heteroresistant subclones and increased sensitivity, while parent bacteria MIC remained unchanged. Indirectly, *mepA* overexpression may be the main cause of heteroresistance, suggesting its role in the potential drug-inhibiting ciprofloxacin heteroresistance phenotype. Additionally, many reports link changes in membrane potential to antibiotic resistance [22,26,36]. This study found that the membrane potential in 529_HR was lower than in 529, which was possibly caused by the downregulation of 529_HR membrane potential related genes (*qoxA*, *qoxB*, *qoxC*, *qoxD*, *mprF*). In *S. aureus*, mutations in these genes reduce membrane potential and gentamicin lethal activity [37]. Heteroresistant subclones may produce heteroresistance phenotypes by decreasing membrane potential and metabolic activity, suggesting that decreased membrane potential also contributes to heteroresistance.

## 4. Materials and Methods

### 4.1. Bacterial Strains

*S. aureus* GD18_SA_529, GD18_SA_284, and GD18_SA_480 (hereafter referred to as 529, 284, and 480, respectively) methicillin-resistant *S. aureus* isolates from the Guangdong Provincial Center for Disease Control and Prevention were used as test strains in this study. *S. aureus* ATCC29213 and *E. coli* ATCC25922 served as quality control strains and are listed in Table S1. Institutional Review Board or Institutional Animal Care and Use Committee approval was not required because our research did not involve human subjects or animal experimentation.

### 4.2. Antimicrobial Susceptibility Testing

The minimum inhibitory concentrations (MIC) were determined using the broth microdilution method per the European Committee on Antimicrobial Susceptibility Testing (EUCAST) guidelines for the following antibiotics: ciprofloxacin, tigecycline, vancomycin, meropenem, rifampicin, tobramycin, gentamycin, amikacin, linezolid, erythromycin, ampicillin, and trimethoprim/sulfamethoxazole [38,39,40]. The testing was performed in triplicate. *S. aureus* ATCC29213 and *E. coli* ATCC25922 were employed as quality control strains. The minimum bactericidal concentration (MBC) was determined using a modified broth microdilution method. Briefly, after obtaining the MIC, aliquots (100 μL) from wells showing no visible bacterial growth were subcultured onto fresh agar plates. After 24 h of incubation, MBC was recorded as the lowest concentration with ≤10 colony-forming units (CFUs).

### 4.3. Population Analysis Profiling

Population analysis profiling (PAP) was used to detect ciprofloxacin heteroresistance using standard published techniques [41]. Briefly, aliquots of 100 μL of 10-fold serially diluted overnight cultures of isolate were spread evenly on Mueller–Hinton (MH) agar plates containing ciprofloxacin at 0×, 1/2×, 1×, 2×, 4×, 8×, and 16× MIC. After overnight incubation at 37 °C, colonies were counted to calculate CFU/mL. The experiments were repeated three times.

### 4.4. Stability Evaluations and Growth Curve

The parental strain and the heteroresistant subclone (*S. aureus* GD18_SA_529_HR, referred to as 529_HR) were passaged on antibiotic-free MH agar plates every 24 h for 7 days, and the MICs were determined daily to monitor the stability of the phenotype [42]. Briefly, aliquots of bacterial suspension prepared from an overnight Luria–Bertani (LB) broth culture were 1000-fold diluted in 50 mL LB broth. Cultures were shaken at 180 rpm at 37 °C for 24 h, and the absorbance at 600 nm (OD_600_) was measured at 0, 2, 4, 6, 8, 10, 12, and 24 h. Each isolate was tested in triplicate [43].

### 4.5. Whole-Genome Sequence and Sequence Analysis

Whole-genome DNA from the 529 parental strain and heteroresistant subclone was extracted using a TIANamp Bacterial DNA Kit (TIANGEN, Beijing, China). WGS was performed on the Illumina NovaSeq 6000 platform (Illumina, San Diego, CA, USA) with 2 × 150 bp paired-end reads. SPAdes (v. 3.15.5) was used for genome assembly, and QUAST (v. 5.2) was used to assess the assemblies for quality metrics [44,45]. All assembled genomes were annotated using Prokka (v. 1.14.6) [46]. Resistance genes and plasmid replicon types were identified based on a comparison with the ResFinder and PlasmidFinder database [46,47]. Snippy (v. 4.6.0) (https://github.com/tseemann/snippy, accessed on 29 October 2023) was used to compute the genome alignment of strains, using *S. aureus* NCTC8325 as the reference sequence. The functions of differential mutant gene products were described by Gene Ontology (GO analysis; http://geneontology.org, accessed on 20 March 2023) [48].

### 4.6. RNA-seq and Transcriptomic Analysis

RNA from the 529 parental strain and heteroresistant subclone was extracted using OMEGA Bacteria RNA Kit (Omega Bio-tek, Norcross, GA, USA). RNA integrity was determined, and the mRNA library was constructed and sequenced on Illumina NovaSeq 6000 with 2 × 150 bp paired-end reads. Quality control and data filtering was performed using Fastp (v. 0.23.2) [49]. Reads were mapped to a *S. aureus* NCTC8325 mRNA reference sequence using FANSe2, and the parameters were L-160 E-11 I-1 S-14; genes with <10 mapped reads were considered unreliable and removed [50]. The mRNAs were normalized using reads per kilobase per million mapped reads (RPKM) [51]. Differential expression analysis used R (v. 4.3.1) with the edgeR (v. 3.42.4) package; cutoffs were Log_2_ fold change ≥ 1 or ≤−1 and *p*-value corrected for multiple guessing ≤ 0.05 [52]. Gene Ontology Enrichment Analysis of differentially expressed genes was conducted via Gene Ontology (GO analysis; http://geneontology.org, accessed on 20 March 2023) [48].

### 4.7. Determination of Limonene Inhibitory Effectiveness

The MICs of ciprofloxacin at different limonene concentrations were determined by checkerboard assay. In brief, each microplate well received 1 μL of working inoculum and 99 μL of MH broth medium. The wells were filled with 50 μL of ciprofloxacin 2-fold dilutions ranging in concentration from 1/32× MIC to 2× MIC and 50 μL of limonene 2-fold dilutions ranging in concentration from 0 mg/L to 2560 mg/L. After 12 h of incubation at 37 °C, OD_600_ was measured on the cultures [53]. An optimal concentration of limonene and varying amounts of diluted ciprofloxacin were added into MH agar plates. Aliquots of 100 μL of 10-fold serially diluted overnight cultures of isolates shaken at 180 rpm at 37 °C were equally disseminated on appropriate agar plates, and CFUs were calculated as previously described.

### 4.8. DiOC_2_(3) Membrane Potential Measurement

The exponential phase (OD_600_ of 0.4) of the parental strain and heteroresistant subclone were pelleted by centrifugation at 5000× *g* for 5 min to remove the growth medium. The cells were washed with 1× phosphate-buffered saline (PBS), then centrifuged, and resuspended. Then, 1 mM 3,3′-Diethyloxacarbocyannine iodide [DiOC_2_(3)] was added to the cells for a final concentration of 10 μM. A 96-well opaque microplate was filled with aliquots of 180 μL DiOC_2_(3)-loaded cells, then incubated for 20 min without light exposure. Cultures were quantified by measuring fluorescence (emission at 670 nm and excitation at 450 nm) every 2 min for 1 h. Ciprofloxacin was diluted and added to wells for 10 min at varying concentrations: 0.06 mg/L, 0.125 mg/L, 0.25 mg/L, 0.5 mg/L, 1 mg/L, and 2 mg/L. For disruption of ∆φ, carbonyl cyanide m-chlorophenyl hydrazine (CCCP) was used. Each isolate was tested three times, and the propensity of membrane potential to drop was validated by detecting the fluorescence of cells treated with 0.5 mg/L ciprofloxacin [54]. Aliquots of 100 μL of 10-fold serially diluted cultures of 0 h and 1 h DiOC_2_(3)-loaded cells were evenly spread on MH agar plates, and the CFU/mL was calculated as previously described.

### 4.9. RT-qPCR

The Vazyme HiScript III All-in-one RT SuperMix (Vazyme, Nanjing, China) was used to reverse transcribe the RNA sample, and quantitative reverse transcription-PCR (qRT-PCR) was performed in triplicate using gene-specific primers (Table S3), the Vazyme Taq Pro Universal SYBR qPCR Master Mix (Vazyme, Nanjing, China), and the Roche LightCycler 96 system (Roche, Basel, Switzerland). The initial denaturation for 30 s at 95 °C was followed by 40 cycles of denaturation for 10 s at 95 °C, annealing for 30 s at 60 °C, and extension for 30 s at 72 °C during PCR amplification [55].

## 5. Conclusions

To our knowledge, this is the first report of ciprofloxacin heteroresistance in *S. aureus* strains in China. Upregulation of *mepA* efflux pumps and a reduction in membrane potential may be the main heteroresistance mechanisms. Additionally, limonene might be a potentially effective agent to inhibit ciprofloxacin heteroresistance phenotypes.

## Figures and Tables

**Table 1:** Resistance genes and efflux pumps of *S. aureus* GD18_SA_529.

	Type	Gene	Substrate
Resistance genes	Beta-lactams	*blaZ*	Methicillin
	Macrolides	*erm(A)*	Erythromycin
	Aminoglycosides	*spc*	Spectinomycin
	Rifamycins	*rpoB*	Rifampicin
Multidrug resistant efflux pumps	MFS family	*norA*, *norB*, *norG*	Ciprofloxacin
		*sdrM*	Norfloxacin Acriflavine Ethidium bromide
	MATE family	*mepA*	Ciprofloxacin Norfloxacin Tigecycline

**Table 2:** SNPs between *S. aureus* GD18_SA_529 and GD18_SA_529_HR.

SNP Position	Gene	SNPs
188,068	*ggt*	p.Val549Ser
294,112	*essG_6*	p.Leu7Gln
390,715	*ssl3*	p.Ile157Thr
390,730		p.ThrAspMetThr162LysGluIleAsn
798,468	*ssp*	p.Gln200_Ser201dup
1,043,951	*isdB*	p.Glu71_Thr72delinsValAlaLysProValAla
1,338,822	*asd*	p.Asp242Glu
1,378,171	*ebh*	p.Asp8843Gly
1,684,208	*lysP_2*	p.Glu375Gln
1,684,220		p.Lys371Glu
2,246,711	*farB_1*	p.Thr253Ser
2,723,613	*clfB*	p.Asp663Glu

## Data Availability

The datasets presented in this study can be found in online repositories. The raw data of 529 and 529_HR from WGS and RNA-seq are available in the NCBI Sequence Read Archive repository under BioProject ID PRJNA1051287 (accession numbers: SRR27185055 and SRR27185054) and PRJNA1051527 (accession numbers: SRR27185756 to SRR27185761).

## Supplementary Materials

The following supporting information can be downloaded at: https://www.mdpi.com/article/10.3390/ijms26052372/s1.
