# Supplementary material for: Mechanisms of mepA Overexpression and Membrane Potential Reduction Leading to Ciprofloxacin Heteroresistance in a Staphylococcus aureus Isolate
Source: Int J Mol Sci. 2025 Mar 6;26(5):2372. doi: 10.3390/ijms26052372 (PMC11901101; doi:10.3390/ijms26052372)

Supplemental Materials for

Mechanisms of *mepA* overexpression and membrane potential reduction leading to ciprofloxacin heteroresistance in a *Staphylococcus aureus* isolate

Mengyuan Li[1,2,3†], Qianting Jian[1,2,3†], Xinyi Ye [1,2,3], Mou Jing[1,2,3], Jia'en Wu[1,2,3], Zhihong Wu[1,2,3], Xiaoling Long [1,2,3], Xiaoling Long[1,2,3], Rongmin Zhang[1,2,3], Hao Ren[1,2,3], Jian Sun[1,2,3], Yahong Liu[1,2,3], Xiaoping Liao[1,2,3*], Xinlei Lian[1,2,3*]

[1]State Key Laboratory for Animal Disease Control and Prevention, South China Agricultural University, Guangzhou, China

[2]National Risk Assessment Laboratory for Antimicrobial Resistance of Animal Original Bacteria, South China Agricultural University, Guangzhou, China

[3]Guangdong Provincial Key Laboratory of Veterinary Pharmaceutics Development and Safety Evaluation, South China Agricultural University, Guangzhou, China.

*Corresponding author:

Dr. Xinlei Lian, College of Veterinary Medicine, South China Agricultural University, Guangzhou, E-mail: xinlei_lian@scau.edu.cn

Prof. Xiaoping Liao, College of Veterinary Medicine, South China Agricultural University, Guangzhou, E-mail: xpliao@scau.edu.cn

[†]These authors contributed equally to this work.

Supplementary Tables

Table S1. Ciprofloxacin MIC of bacterial strains used in the current study (mg/L).

**Strain**	**MIC**
S. aureus ATCC29213	0.25
S. aureus GD18_SA_529	0.125
S. aureus GD18_SA_529_HR	1
S. aureus GD18_SA_284	0.25
S. aureus GD18_SA_480	16
E. coli ATCC25922	0.004

Table S2. Ciprofloxacin MBC of *S. aureus* GD18_SA_529 and GD18_SA_529_HR (mg/L).

**Strain**	**MBC**
S. aureus GD18_SA_529	0.5
S. aureus GD18_SA_529_HR	2

Table S3. Primers used for RT-qPCR.

Primers	Sequence (5′-3′)
16S-F	ATTAGATACCCTGGTAGTCCACGC
16S-R	TTGCGGGACTTAACCCAAC
mepA-F	ACACTGCCGATATTTGCCAT
mepA-R	GCTAATGTTTCGCCTTTTGCTC
qoxA-F	AGCACGTGATGTTGCATTCC
qoxA-R	CCTGCTAGTGGCGCATAGTT
qoxB-F	CCTGCTAGTGGCGCATAGTT
qoxB-R	AGCACGTGATGTTGCATTCC
qoxC-F	GCATGGTTGTCACGTATCGC
qoxC-R	GTCTAATCCGCGTCGTTGGA

Supplementary Figures

Figure S1. The chromosomal DEGs GO enrichment analysis of (A) biological processes and (B) molecular functions for 529_HR vs. 529.

A

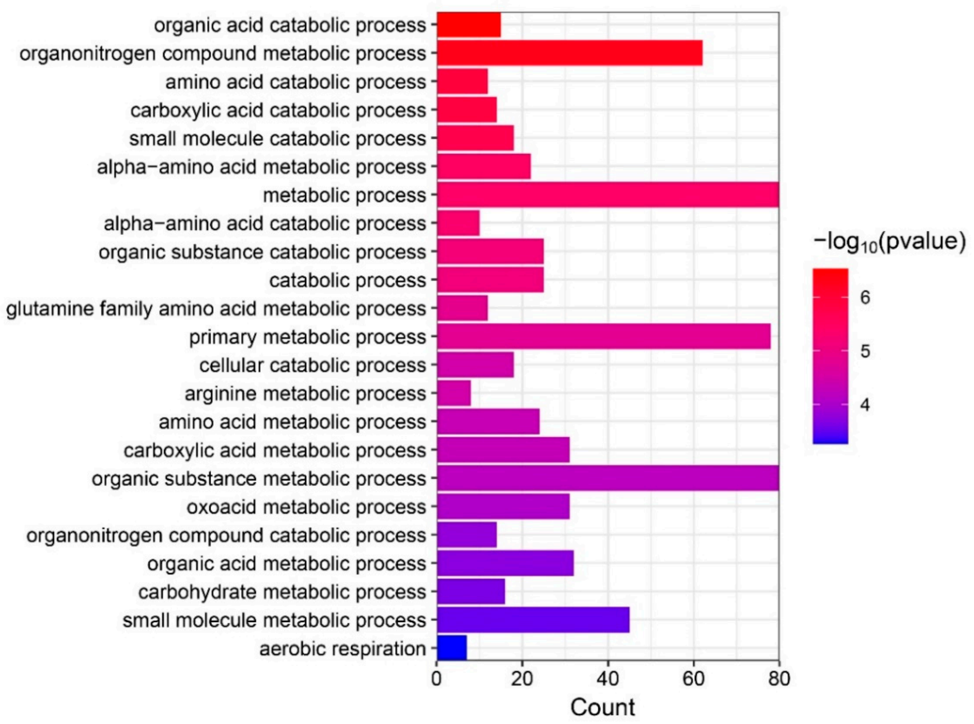

B

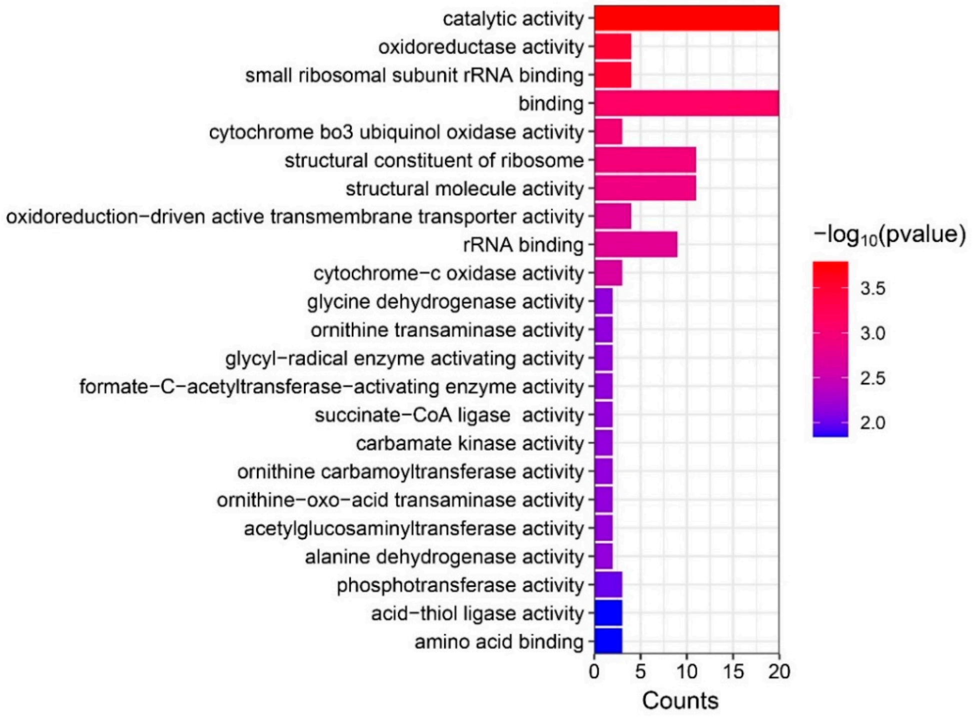

Figure S2. Regulation of genes linked to membrane potential (*mepA*, *mprF*, *qoxA*, *qoxB*, *qoxC*, *qoxD*).

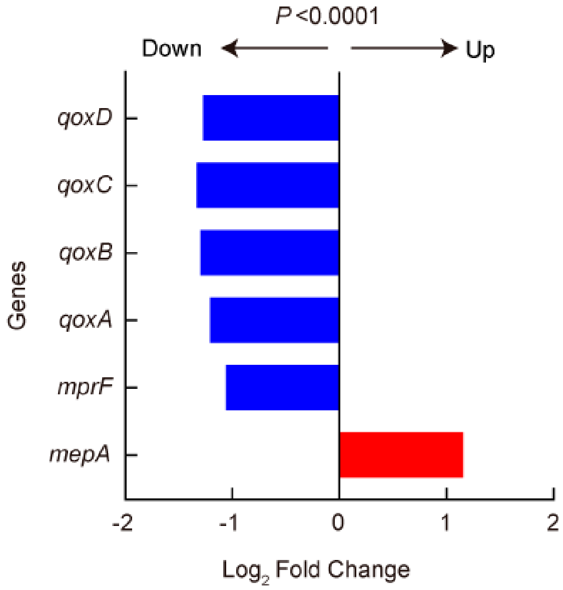

Figure S3. RT-qPCR validated *mepA*, *qoxA*, *qoxB*, and *qoxC* expression level changes in 529_HR relative to 529.

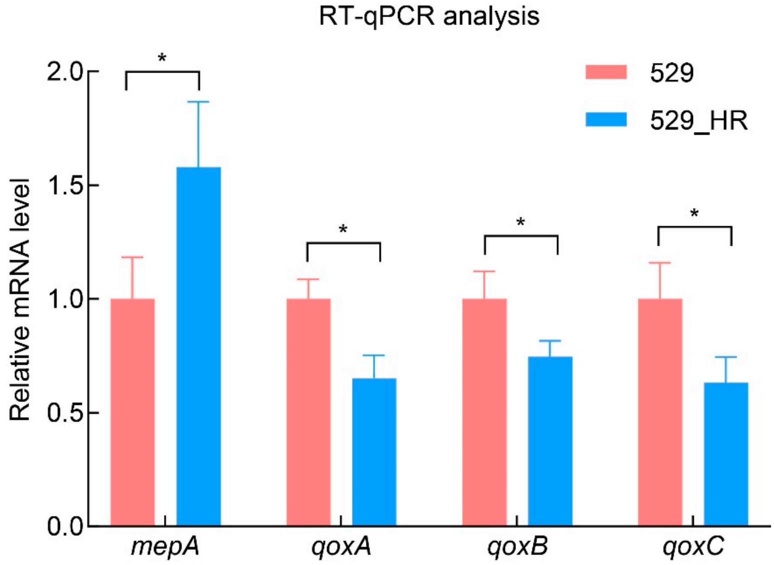

Figure S4. Strains 529 and 529_HR were continuously monitored for fluorescence for 1 h in the presence or absence of CCCP and with varying concentrations of ciprofloxacin.

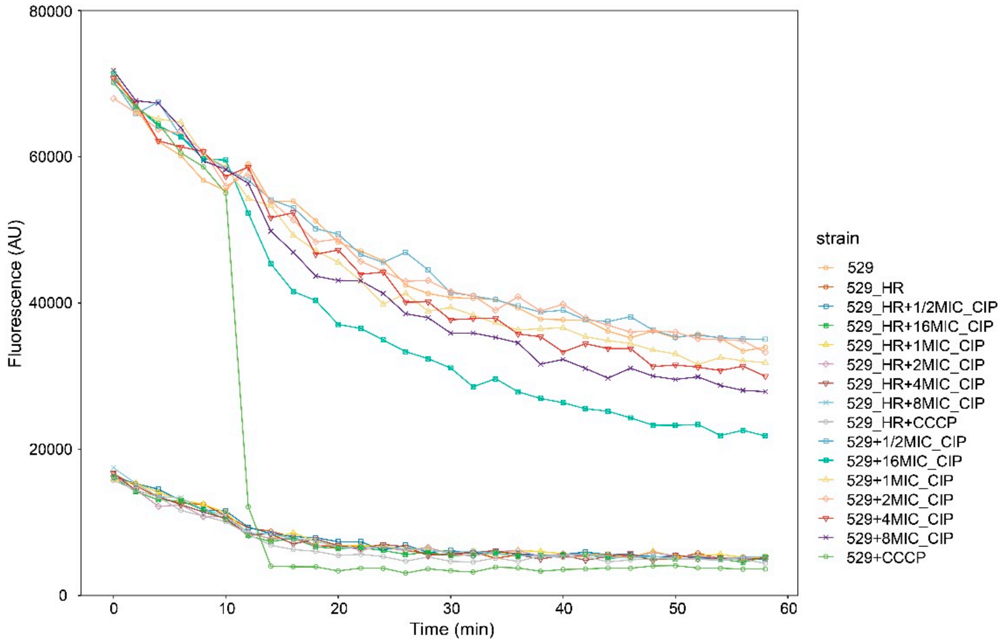

Supplement: Supplementary file 1 [file ijms-26-02372-s001.zip › ijms-3497727-supplementary.pdf]
